# Childhood Sports Participation Is Associated With Health-Related Quality of Life in Young Men: A Retrospective Cross-Sectional Study

**DOI:** 10.3389/fspor.2021.642993

**Published:** 2021-04-22

**Authors:** Kaija Appelqvist-Schmidlechner, Heikki Kyröläinen, Arja Häkkinen, Tommi Vasankari, Matti Mäntysaari, Tuomas Honkanen, Jani P. Vaara

**Affiliations:** ^1^Finnish Institute for Health and Welfare, Mental Health Unit, Helsinki, Finland; ^2^Centre for Military Medicine, Helsinki, Finland; ^3^Faculty of Sport and Health Sciences, University of Jyväskylä, Jyväskylä, Finland; ^4^Department of Leadership and Military Pedagogy, National Defence University, Helsinki, Finland; ^5^Health Sciences, University of Jyväskylä, Jyväskylä, Finland; ^6^Department of Physical Medicine and Rehabilitation, Central Hospital of Central Finland, Jyväskylä, Finland; ^7^Urho Kaleva Kekkonen Institute for Health Promotion, Tampere, Finland; ^8^Faculty of Medicine and Health Technology, Tampere University, Tampere, Finland

**Keywords:** sports participation, health-related quality of life, mental health, physical activity, leisure time, childhood, men, quality of life

## Abstract

The aim of the study was to examine whether sports participation (SP), engagement in competitive sports (CS), and the type of sport undertaken at the age of 12 are associated with the physical and mental components of health-related quality of life (HRQoL) in young adulthood. The data were collected using questionnaires prior to a compulsory military refresher training course in Finland. The sample consisted of 784 men (mean age 26 years). HRQoL was measured with RAND 36 and childhood SP with a series of questions. Data were analyzed with logistic regression. Higher frequency of SP, participation in district-level CS; performing team, endurance, or extreme sports; and playing yard games in childhood were after adjustments all associated with better HRQoL in early adulthood. The association was mainly found with the mental component, and to a lesser extent with the physical component, of HRQoL. Team (OR 1.43, CI 1.00–2.06) and extreme sports (OR 1.77, CI 1.19–2.63) were associated with better mental HRQoL, while playing yard games (OR 0.62, CI 0.44–0.89) reduced the likelihood for having low physical HRQoL. SP in childhood—in the forms of team or individual sports, but also as informal physical activity, such as playing yard games—is associated with HRQoL in young adulthood.

## Introduction

The positive impact of physical activity (PA) on physical and mental health as well as on health-related quality of life (HRQoL) has been well-established (Biddle, 2016; Warburton and Bredin, 2017; Wu et al., 2017; Bize et al., 2018; Marker et al., 2018). HRQoL is a multidimensional concept that includes physical, mental, emotional, and social functioning (Ferrans, 2005). On the individual level, HRQoL includes perceptions of physical and mental health status. It encompasses the perceived health attributes such as the sense of comfort or well-being, and the ability to maintain good physical, emotional, and cognitive functions including the ability to take part in social activities (Bize et al., 2018). The concept of HRQoL has gained attention in the past few decades, as it has been found to be a stronger predictor of mortality and morbidity than many other objective measures of health (Dominick et al., 2002; DeSalvo et al., 2006). Finding determinants and predictors of HRQoL may help to prevent diseases and disabilities and to promote general well-being in different populations. HRQoL is known to associate with various socioeconomic factors and variables related to health behavior, such as body mass index (BMI), use of alcohol, smoking, and PA (Wu et al., 2017; Bize et al., 2018; Marker et al., 2018; Ellina et al., 2019).

Participation in sports during leisure time leads to various psychological, social, and health benefits both in adults (Eime et al., 2013b) and in children (Eime et al., 2013a). Furthermore, previous research has provided evidence of a positive association between sports participation and HRQoL in children (Moeijes et al., 2019a,b), adolescents (Snyder et al., 2010), university students (Shaikh et al., 2016), and women (Eime et al., 2010). However, there is a lack of evidence on the association of childhood sports participation and HRQoL in adulthood. Few studies with a longitudinal study design have investigated this relationship, but commonly in children and with a relatively short follow-up periods of between 1 and 2 years (Vella et al., 2014; Moeijes et al., 2019a). Stracciolini et al. (2018) investigated the association between sports participation in college and HRQoL in middle age and older adulthood. They found that participation in collegiate sports was associated with positive health outcomes in later life. However, association was also found with increased anxiety in older age.

Associations between different levels and types of sports participation and HRQoL have not been widely studied. The study by Moeijes et al. (2019b) found that particularly outdoor sports, rather than indoor sports, were significantly associated with better HRQoL in children. The association seemed to be present especially in the physical domain of HRQoL and to a lesser degree in the psychological domain of HRQoL. Similarly, the systematic reviews by Eime et al. (2013a,b) found that sports participation has many different psychological, social, and health benefits both in adult populations as well as in children and adolescents. Particularly, club-based or team-based sports, rather than individual activities, are associated with improved health outcomes (Eime et al., 2013a,b). However, there is insufficient evidence regarding the relationship between sports participation and HRQoL and lack of knowledge on the impact of participating in different sport types in childhood on HRQoL in adulthood.

Evidence on HRQoL outcomes of competitive sports across the life span is scarce. Previous research indicates that HRQoL is commonly higher in athletes than in non-athletes (Houston et al., 2016). In other respects, this topic has been most commonly investigated from the perspective of physical, mental, or psychosocial health rather than directly from the perspective of HRQoL (Backmand et al., 2009; Sorensen et al., 2014; Appelqvist-Schmidlechner et al., 2018). From a longitudinal perspective, some evidence is provided by a study among former Finnish elite athletes (Backmand et al., 2009) and the study of Sorensen et al. (2014), indicating some physical health concerns but better psychosocial health among elite intercollegiate student-athletes compared with non-athletes in a life-span perspective. In our previous study (Appelqvist-Schmidlechner et al., 2018) with the same sample of young men, competitive sports in childhood were associated with better mental health in adulthood. However, to the best of our knowledge, there is no previous research about the impact of participation in competitive sports in childhood on HRQoL in young adulthood.

The aim of the present study was to investigate retrospectively the association between sports participation in childhood and HRQoL in young adulthood among young Finnish men from the perspective of physical and mental aspects of HRQoL. The study aimed to explore this association in terms of (1) frequency of organized sports participation, (2) the role and level of participation in competitive sports, and (3) the type of sport at the age of 12 years. Despite partly contradictory findings of previous studies, we hypothesized that higher frequency and level of sports participation in childhood would be associated with higher HRQoL in adulthood. Furthermore, especially group-based sports were expected to be associated with higher HRQoL.

## Materials and Methods

The study participants were young adult men (mean ± SD age 26 ± 7 years) who were called up to the military refresher training organized by the Finnish Defence Forces. In Finland, the Defence Forces are based on a universal male conscription; and each year, 70–75% of all young Finnish men (about 20,000 men) perform their military service. After the military service, they can be called up to a military refresher training lasting 4–10 days as reservists.

The study participants were informed about the study in the military refresher training call-up letter. The data were gathered with a self-administered questionnaire at the beginning of seven military refresher training courses that were carried out in 2015 in different counties around Finland. Participation in the study was voluntary, and of 823 course participants, 792 participated in the study. All participants signed a written consent form. The sample of this study consisted of all male participants (*n* = 784). Due to some missing values in the outcome variables, the total study sample was 777.

The study was part of the Finnish Reservist 2015 study that aimed to investigate the physical performance and health of Finnish reservists. The study was approved by the ethical committees of the Central Finland Health Care District and the Headquarters of the Finnish Defence Forces (AM5527). The research was conducted in the absence of any commercial or financial relationships that could be construed as a potential conflict of interest.

### Measurements

HRQoL was measured using the Finnish RAND 36-item health survey (Aalto et al., 1995). It contains eight dimensions that can be aggregated into two summary scores: the physical (including physical functioning, physical role functioning, bodily pain, and general health) and mental (including emotional role functioning, vitality, mental health, and social role functioning) component summary scores. The reliability and validity of the scale have been reported to be good (Cronbach's alpha coefficient between 0.80 and 0.94) (Aalto et al., 1995). The responses were given in a six-point scale. First, numeric values were recoded per the scoring key and transformed into a 0–100 scale, with higher scores indicating higher HRQoL. Then, items in the same dimensions were averaged together to create scores for the eight dimensions and main component of the scale. For further analysis, in order to see if relations are different in different parts of the response distribution, the scores were divided into three tertiles (low, moderate, and high levels of HRQoL) for the physical and mental components of RAND 36. The scores within physical component were ≤ 86.88 for low, 86.89–93.02 for moderate, and ≥93.03 for high and within mental component ≤ 78.88 for low level, 78.89–89.75 for moderate level, and ≥89.76 for a high level of HRQoL.

Participation in sports in childhood was measured with one single question “How often did you participate in training or other structured sports activity at the age of 12 years?” The responses were 1 = not at all, 2 = once a month, 3 = 2–3 times a month, 4 = 1–2 times a week, 5 = 3–4 times a week, and 6 = 5 times a week or more. For the analysis, responses 2 and 3 were combined, as both responses indicate a similarly low level of participation. Study participants were asked to choose from the list of all the types of sports, which they performed regularly (weekly) at the age of 12 years. The list included the following types of sports (with examples of types of sports in parentheses): team sport (football, ice hockey, basketball, etc.), endurance sport (running, orienteering, swimming, cycling, etc.), strength sport (gym, cross fit, weightlifting, etc.), sports that require technical performance (golf, gymnastics, table tennis, etc.), extreme sport (climbing, diving, surfing, and alpine skiing), combat sport (wrestling, boxing, judo, karate, etc.), and playing yard games.

Participation in competitive sports in childhood was measured with the question “If you participated in competitive sports in childhood, at which level did you compete?” The responses were 1 = I did not participate in competitive sports, 2 = school, 3 = sports club, 4 = district, 5 = national, and 6 = international. For the analysis, responses 5 and 6 were combined.

Self-reported leisure-time PA (LTPA) in adulthood was determined from responses to a single question “Which of the following definitions best describe your leisure-time physical activity habits? (Think of the last 3 months and consider all leisure-time physical activities that lasted at least 20 min per session)” with six response categories: 1 = less than once a week; 2 = no vigorous activities, but light or moderate PA at least once a week; 3 = brisk PA once a week; 4 = vigorous activity twice a week; 5 = vigorous activity three times a week; and 6 = vigorous activity at least four times a week. The question has been validated against fitness (Fogelholm et al., 2006) but not against other instruments measuring PA. For further analysis, groups for inactive (less than once a week), low (light or moderate PA at least once a week), medium (vigorous PA once to three times a week), and high (vigorous activity at least four times a week) activity groups were computed to distinguish four clearly different groups in terms of PA.

The questionnaire also included sociodemographic background (including age, educational level, employment, and marital status) and health behavior (smoking and use of alcohol). Body height and weight were measured, and BMI was calculated and classified into four categories: underweight <18.50, normal 18.50–24.99, overweight 25.00–29.99, and obesity/severe obesity ≥ 30.

### Statistics

The descriptive data of the study sample are presented in Table 1. The normality of distribution of RAND 36 was tested by Shapiro–Wilk test, and the data proved non-normally distributed. The mean scores for combined physical and mental component scores of RAND 36 were grouped together by variables describing the sports participation of the study participants at the age 12 (Table 2). The statistical significance of the RAND 36 score differences between the groups was calculated using the non-parametric Kruskal–Wallis and Mann–Whitney tests, as the data were not normally distributed. The association between sports participation and HRQoL, in terms of physical and mental component scores of RAND 36, was then explored with the help of logistic regression analyses. Before regression analysis, Spearman's rank correlation coefficient was used to give an indication of the magnitude of association (collinearity) between explanatory variables. Odds ratios and 95% confidence intervals (CIs) were calculated in the physical and mental components of HRQoL for each group. Unadjusted and fully adjusted models are presented for low compared with moderate and high HRQoL (Table 3) and for high compared with moderate and low HRQoL (Table 4), as the focus of interest was to detect if the relations are different in the different parts of the response distribution. Age, educational level (primary, secondary, and high school), employment status (employment or education/not in employment or education), marital status (married or cohabitation/single), the present LTPA (inactive/low/medium/high), use of alcohol (not using/once a month or less often/about twice a month/1–2 a week/3–4 a week/5–6 a week or daily), smoking, and BMI (categories 1–4 presented above) were used as covariates in the fully adjusted model. The level of statistical significance was set to *p* < 0.05. Analysis was performed with IBM SPSS Statistic 26 program.

**Table 1:** Characteristics of the study population.

**Variable**	**Mean/frequency (%)**
Age (*n* = 777)	26 (SD 7) years
**Marital status (*****n*** **=** **777)**	(%)
Married/cohabiting	55
Single	45
**Education (*****n*** **=** **776)**	
Comprehensive school	6
Vocational school	42
High school degree	30
Upper secondary school or academic degree	23
**Employment status (*****n*** **=** **755)**	
Employment/studying	71
Not in employment or education	29
**Leisure-time physical activity (*****n*** **=** **777)**	
Inactive (less than once a week)	12
Low (light or moderate physical activity at least once a week)	18
Medium (vigorous physical activity 1–3 times a week)	49
High (vigorous activity at least four times a week)	21
**BMI (*****n*** **=** **754)**	
<18.5	2
18.50–24.99	52
25–29.99	34
≥30	12
**Smoking (*****n*** **=** **760)**	
Yes	32
No	68
**Use of alcohol (*****n*** **=** **777)**	
Not using	7
Once a month or less often	17
About twice a month	22
Once or twice a week	35
3–4 times a week	13
5–6 times a week or daily	6

*BMI, body mass index*.

**Table 2:** Mean scores of the physical and mental components of HRQoL according to sports participation at the age of 12 years.

**Variable**	**%**	**Mean score physical component**	p^*^	**Mean score mental component**	p^*^
**Participating in organized sports (*****n*** **=** **777)**					
No participating	25	87.53		77.61	
1–3 times a month	11	86.82		78.14	
1–2 times a week	29	87.85		80.78	
3–4 times a week	26	88.83		82.81	
At least 5 times a week	10	88.37	ns	84.27	<0.001
**Participating in competitive sports (*****n*** **=** **776)**					
No competitive sports	34	87.73		78.39	
At school level	9	87.86		80.78	
At sports club level	22	87.48		81.19	
At district level	27	88.82		82.31	
At national or international level	9	87.53	ns	81.89	0.013
**Participating in team sports (*****n*** **=** **777)**					
Yes	66	88.58		82.02	
No	34	86.91	0.003	77.63	0.001
**Participating in endurance sports (*****n*** **=** **777)**					
Yes	35	89.08		81.92	
No	65	87.43	0.008	79.73	ns
**Participating in strength sports (*****n*** **=** **777)**					
Yes	5	88.50		83.49	
No	95	87.98	ns	80.37	ns
**Participating in sports that requires technical performance (*****n*** **=** **777)**					
Yes	11	88.05		81.70	
No	89	87.99	ns	80.41	ns
**Participating in extreme sports (*****n*** **=** **777)**					
Yes	20	88.83		84.81	
No	80	87.81	ns	79.48	<0.001
**Participating in combat sports (*****n*** **=** **777)**					
Yes	16	86.63		79.82	
No	84	88.27	ns	80.63	ns
**Playing yard games (*****n*** **=** **777)**					
Yes	69	88.61		81.88	
No	31	86.65	0.006	77.42	0.004

*ns, non-significant differences between the groups; HRQoL, health-related quality of life*.

* *Kruskal–Wallis or Mann–Whitney U-tests*.

**Table 3:** Odds ratios (OR) and 95% confidence intervals (CIs) separately for low compared with moderate and high scores in the physical and mental component summary of RAND 36 by variables describing sports participation at age 12 years.

	**Low compared with moderate and high HRQoL**				**Low compared with moderate and high HRQoL**			
	**Physical component**				**Mental component**			
	**Unadjusted**		**Fully adjusted**^****a****^		**Unadjusted**		**Fully adjusted**^****a****^	
	**OR**	**CI (95%)**	**OR**	**CI (95%)**	**OR**	**CI (95%)**	**OR**	**CI (95%)**
**Participation in organized sports**								
No participating	Ref.		Ref.		Ref		Ref.	
1–3 times a month	1.27	0.75–2.16	1.69	0.95–2.99	0.82	0.48–1.40	0.92	0.52–1.62
1–2 times a week	0.77	0.51–1.16	0.83	0.53–1.31	0.56	0.37–0.85^**^	0.60	0.38–0.94^*^
3–4 times a week	0.75	0.49–1.14	0.95	0.59–1.53	0.50	0.32–0.77^**^	0.54	0.34–0.89^*^
At least 5 times a week	0.70	0.39–1.24	0.95	0.50–1.78	0.56	0.31–0.99^*^	0.65	0.35–1.20
**Participation in competitive sports**								
No competitive sports	Ref.		Ref.		Ref.		Ref.	
At school level	0.89	0.50–1.57	1.09	0.59–2.00	0.73	0.41–1.30	0.70	0.39–1.28
At sports club level	0.97	0.66–1.45	0.96	0.62–1.51	0.66	0.43–1.01	0.62	0.39–0.98^*^
At district level	0.72	0.48–1.06	0.85	0.54–1.32	0.63	0.42–0.94^*^	0.70	0.45–1.09
At national or international level	0.93	0.53–1.63	1.21	0.65–2.26	0.68	0.38–1.22	0.76	0.41–1.43
**Participating in team sports**								
No	Ref.		Ref.		Ref.		Ref.	
Yes	0.66	0.48–0.90^**^	0.77	0.54–1.09	0.66	0.48–0.91^*^	0.76	0.54–1.08
**Participating in endurance sports**								
No	Ref.		Ref.		Ref.		Ref.	
Yes	0.71	0.52–0.99^*^	0.84	0.59–1.20	0.72	0.52–1.00^*^	0.81	0.57–1.15
**Participating in strength sports**								
No	Ref.		Ref.		Ref.		Ref.	
Yes	0.70	0.33–1.48	0.63	0.28–1.43	0.50	0.21–1.16	0.45	0.18–1.11
**Participating in sports that requires technical performance**								
No	Ref.		Ref.		Ref.		Ref.	
Yes	0.89	0.55–1.45	0.90	0.52–1.54	0.76	0.46–1.27	0.70	0.40–1.22
**Participating in extreme sports**								
No	Ref.		Ref.		Ref.		Ref.	
Yes	0.80	0.54–1.18	0.84	0.55–1.28	0.59	0.39–0.90^*^	0.61	0.40–0.96^*^
**Participating in combat sports**								
No	Ref.		Ref.		Ref.		Ref.	
Yes	1.24	0.83–1.86	1.12	0.72–1.74	1.15	0.76–1.74	119	0.76–1.86
**Playing yard games**								
No	Ref.		Ref.		Ref.		Ref.	
Yes	0.60	0.43–0.83^**^	0.62	0.44–0.89^**^	0.66	0.48–0.92^*^	0.75	0.53–1.07

a *Adjusted for age, educational level, marital status, employment status, leisure-time physical activity, smoking, use of alcohol, and body mass index (BMI)*.

* *p < 0.05*,

** *p < 0.01*.

**Table 4:** Odds ratios (OR) and 95% confidence intervals (CIs) separately for high compared with low and moderate scores in the physical and mental component summary of RAND 36 by variables describing sports participation at age 12 years.

	**High compared with low and moderate HRQoL**				**High compared with low and moderate HRQoL**			
	**Physical component**				**Mental component**			
	**Unadjusted**		**Fully adjusted**^****a****^		**Unadjusted**		**Fully adjusted**^****a****^	
	**OR**	**CI (95%)**	**OR**	**CI (95%)**	**OR**	**CI (95%)**	**OR**	**CI (95%)**
**Participation in organized sports**								
No participating	Ref.		Ref.		Ref		Ref.	
1–3 times a month	0.71	0.39–1.29	0.58	0.31–1.10	0.81	0.42–1.55	0.72	0.36–1.44
1–2 times a week	1.09	0.71–1.65	0.92	0.58–1.45	1.62	1.04–2.55^*^	1.62	1.00–2.60^*^
3–4 times a week	1.35	0.88–2.07	1.05	0.66–1.67	2.29	1.46–3.61^***^	2.27	1.39–3.69^**^
At least 5 times a week	1.28	0.72–2.26	0.86	0.51–1.75	2.51	1.41–4.47^**^	2.35	1.27–4.36^**^
**Participation in competitive sports**								
No competitive sports	Ref.		Ref.		Ref.		Ref.	
At school level	1.10	0.62–1.97	0.84	0.45–1.57	1.28	0.71–2.34	1.28	0.69–2.38
At sports club level	0.96	0.63–1.46	0.94	0.60–1.50	1.49	0.96–2.29	1.52	0.96–2.40
At district level	1.25	0.84–1.85	1.04	0.67–1.59	1.92	1.28–2.87^**^	1.83	1.18–2.83^**^
At national or international level	0.93	0.52–1.68	0.70	0.37–1.33	1.28	0.71–2.40	1.25	0.66–2.36
**Participating in team sports**								
No	Ref.		Ref.		Ref.		Ref.	
Yes	1.35	0.97–1.87	1.14	0.80–1.62	1.53	1.09–2.14^*^	1.43	1.00–2.06^*^
**Participating in endurance sports**								
No	Ref.		Ref.		Ref.		Ref.	
Yes	1.37	0.99–1.89	1.20	0.85–1.69	1.15	0.84–1.60	1.07	0.76–1.50
**Participating in strength sports**								
No	Ref.		Ref.		Ref.		Ref.	
Yes	1.54	0.78–3.07	1.76	0.85–3.65	1.96	0.98–3.92	1.87	0.92–3.81
**Participating in sports that requires technical performance**								
No	Ref.		Ref.		Ref.		Ref–	
Yes	1.31	0.81–2.12	1.33	0.80–2.19	1.14	0.70–1.86	1.21	0.73–2.01
**Participating in extreme sports**								
No	Ref.	0.94–2.01	Ref.		Ref.		Ref.	
Yes	1.37		1.30	0.87–1.93	1.71	1.17–2.50^**^	1.77	1.19–2.63^**^
**Participating in combat sports**								
No	Ref.		Ref.		Ref.		Ref.	
Yes	0.85	0.56–1.30	0.95	0.61–1.48	100	0.65–1.53	0.96	0.62–1.50
**Playing yard games**								
No	Ref.		Ref.		Ref.		Ref.	
Yes	1.33	0.95–1.88	1.29	0.90–1.86	1.44	1.02–2.05^*^	1.34	0.93–1.93

a *Adjusted for age, educational level, marital status, employment status, leisure-time physical activity, smoking, use of alcohol, and body mass index (BMI)*.

* *p < 0.05*,

** *p < 0.01*,

*** *p < 0.001*.

## Results

The characteristics of the study population are presented in Table 1. The study participants were on average 26 (SD 7) years old. About half of them (55%) were married or cohabiting and 71% employed or in the school. One third reported that they were inactive or engaged in low levels of LTPA. Approximately half of the participants (49%) reported medium levels, and 21% reported high levels of LTPA. Of note, 33% of the participants were overweight, 12% were obese, 19% used alcohol at least three times a week, and 32% were smokers (Table 1).

A majority of the participants reported that they were engaged in organized sports (75%) and competitive sports (64%) at the age of 12 years (Table 2). Team sports were the most common type of sport in childhood. Participants were more likely to engage in team sport (66%). Two of three participants (66%) reported that they engaged in team sports at the age of 12 years, 35% engaged in endurance sport, and 27% engaged in extreme sport. A majority of respondents (69%) played yard games at this age.

Unadjusted mean scores of RAND 36 by different levels of sport participation at age of 12 years are presented in Table 2. Men who had participated in team (*p* = 0.003) or endurance sports (*p* = 0.008) or played yard games (*p* = 0.006) at the age of 12 years had higher scores in the physical component of HRQoL. Higher frequency of sports participation (*p* < 0.001) and level of competitive sports (*p* = 0.013) as well as participation in team (*p* = 0.001) or extreme sports (*p* < 0.001) or playing yard games (*p* = 0.004) at the age of 12 years were associated with higher scores in the mental component of HRQoL.

Logistic regression analysis was conducted to find associating factors for both low (Table 3) and high levels (Table 4) of HRQoL. In terms of the physical component of HRQoL, no significant association was found between frequency or level of competitive sports in childhood and HRQoL in adulthood. However, the unadjusted logistic regression analysis showed a significant association between participation in team (OR 0.66, CI 0.48–0.90) and endurance sports (OR 0.71, CI 0.52–0.99), as well as in yard games (OR 0.60, CI 0.43–0.83) with a low level of HRQoL. Nonetheless, after confounding factors were adjusted, only association with playing yard games at the age of 12 years remained statistically significant (OR 0.62, CI 0.44–0.89). In terms of a high level of HRQoL in the physical component, no significant associations were found.

Several significant associations were found between childhood sports participation and the mental component of HRQoL in young adulthood. In unadjusted models, a low level of HRQoL was significantly associated with less participation in organized sports (OR 0.56, CI 0.37–0.85); lower levels of competitive sports (OR 0.62, CI 0.42–0.94) and nonparticipation with team (OR 0.66, CI 0.48–0.91), endurance (OR 0.72, CI 0.52–1.00), or extreme sports (OR 0.59, CI 0.39–0.90); or playing yard games (OR 0.66, CI 0.48–0.92). Respectively, a high level of HRQoL in the mental component was significantly associated with higher frequency of participation in organized sports, competitive sports at the district level (OR 0.63, CI 0.42–0.94) and participation in team (OR 1.53, CI 1.09–2.14) or extreme sports (OR 1.71, CI 1.17–2.50) or in yard games (OR 1.44, CI 1.02–2.05). After confounding factors were adjusted, participation in team or endurance sports and playing yard games attenuated to non-significant as predictor for a low level of HRQoL and playing yard games as a predictor for a high level of HRQoL. All other associations remained statistically significant. Higher frequency of sports participation was associated only from the perspective of a high level of HRQoL. Thus, the higher the weekly participation, the higher the odds for having a high level of HRQoL in the mental component in young adulthood. The odds for having a low level of HRQoL were the lowest in the group of men who participated in organized sports once to four times a week, but not more. In terms of competitive sports, the likelihood for having a high level of HRQoL in the mental component of HRQoL in young adulthood was the highest in the group of men who had engaged in district-level competitive sports at age of 12 years (OR 1.83, CI 1.18–2.83).

## Discussion

The findings of the present study showed that participation in organized sports at the age of 12 years was positively associated with the mental component of HRQoL, but no clear association was found with the physical component of HRQoL. A higher frequency of childhood participation was associated with a stronger likelihood for having a high level of HRQoL in young adulthood.

Support for these findings can be found particularly in studies with focus on the association between childhood sports participation and mental health in adulthood (Jewett et al., 2014; Sabiston et al., 2015; Ashdown-Franks et al., 2017; Appelqvist-Schmidlechner et al., 2018). Even though the focus of the present study was HRQoL, previous findings with focus on mental health are relevant, as mental health contributes to the mental component of HRQoL. The mental component of the scale includes—besides mental health itself—emotional and social role functioning as well as vitality, with all fields being determinants or strong associating factors of mental health. In the present study, high frequency of sport participation in childhood seemed to be relevant especially from the perspective of having high HRQoL in the mental component of HRQoL in adulthood. In terms of physical component of HRQoL, childhood sports participation seemed to be less associated with the HRQoL in adulthood or associate only with having a low level of HRQoL in adulthood. Thus, PA in childhood may contribute more for mental component of HRQoL, while the physical component of HRQoL is more dependent on the current status of PA. However, drawing direct links from childhood to adulthood is not possible. The findings allow evidence only for associations, not for causality. No direct link between childhood sports participation and HRQoL in young adulthood can be established.

However, there are several potential mechanisms explaining the association between childhood sports participation and HRQoL in young adulthood, especially in terms of mental component of HRQoL and how childhood sports participation may have potential to contribute to the HRQoL in young adulthood. The mechanism behind this relationship may be related to physical self-perception, self-esteem, life skills, social interaction and connectedness, and opportunity to improve social skills through participation in organized sports (Findlay and Coplan, 2008; Lubans et al., 2016). Organized sport groups can be seen as social catalysts that lead to enhanced involvement and participation (Rutten et al., 2008) contributing to increased HRQoL also in the longer term. Lubans et al. (2016) investigated mechanisms between PA for cognitive and mental health in youth in their systematic review. They identified improvements in physical self-perceptions and enhanced self-esteem as the strongest mechanisms responsible for the positive effects of participation in PA on mental well-being. Similarly, the study by Findley and Coplan (Findlay and Coplan, 2008) found that sports participation is positively related to social skills and self-esteem in children. These aspects may in some degree explain the mechanism between childhood sports participation and mental component of HRQoL in adulthood.

The findings of the present study indicate that competitive involvement in sports in childhood may be beneficial for mental component of HRQoL in young adulthood. No association was found between the physical component of HRQoL and participation in competitive sports at the age of 12 years. To the best of our knowledge, there are no previous studies investigating this relationship. However, this finding is supported by several studies that suggest competitive sports to be beneficial for HRQoL (Houston et al., 2016) and mental health (Appelqvist-Schmidlechner et al., 2018; Dore et al., 2018a; Sneddden et al., 2018). There are several potential underlying mechanisms that may explain this relationship. First, competitive sports may teach children valuable life skills, such as goal setting, commitment, and coping strategies to handle stressful situations, which prepare them for to handle challenges and pressures of daily life also in later life (Merkel, 2013) and in this way contribute especially to the mental component of HRQoL in adulthood. Second, perceived sport competence that results in increased self-esteem may play a mediating role in the relationship between competitive sports and mental component of HRQoL (Wagnsson et al., 2014).

Based on comparisons of unadjusted mean scores of HRQoL, team sports and yard games seemed to be associated with both better physical and mental components of HRQoL. Respectively, endurance sports in childhood associated only with the physical component and extreme sports with the mental component of HRQoL in young adulthood. After confounding variables were adjusted, only team and extreme sports as well as yard games in childhood seemed to be associated with HRQoL in later life, with team and extreme sports affecting the mental component and playing yard games the physical component of HRQoL. Some support from previous research can be found to explain these findings (Downward and Rasciute, 2011; Vella et al., 2014; Sabiston et al., 2015; Dore et al., 2018a,b). Dore et al. (2018b) found that particularly team sports and PA in informal groups—like engagement in yard games—were positively associated with mental well-being in young adulthood. The important role of team sports and opportunity for social interaction through sports will be supported also by several other the studies, especially from the perspective of mental health (Downward and Rasciute, 2011; Eime et al., 2013b; Vella et al., 2014; Sabiston et al., 2015). Eime et al. (2013a) found in their systematic review that, particularly, team sports associate with improved health outcomes compared with individual activities. Social nature of team sport and positive involvement of peers and adults may serve as mechanism in this association. However, also individual sports may benefit psychosocial well-being by enhancing the development of true self-awareness and personal growth (Eime et al., 2013b).

An interesting finding was the positive association between HRQoL and participation in extreme sports—most commonly understood as sport that involves speed, height, a high level of physical exertion, and/or highly specialized gear including sports such as skateboarding, snowboarding, parkour, mountain biking, motocross, and alpine skiing. There is existing evidence supporting the meaningfulness of extreme sports and providing links to positive physical and mental health outcomes (Immonen et al., 2017; Roberts et al., 2018). Immonen et al. (2017) summarized the benefits of action and adventure sports—commonly used as synonym for extreme sports—as “(1) opportunities to fulfill basic psychological needs of autonomy, competence and relatedness, (2) opportunities to overcome challenge, (3) opportunities to experience intense emotions, (4) increased positive psychological outcomes such as resilience, self-efficacy, and positive affect, (5) increased physical activity levels and (6) feelings of connection to nature.” They all have potential to promote mental health in childhood in a way that may bear fruit also in young adulthood in terms of mental component of HRQoL. Action and adventure sports present an interesting method for sport-based interventions that can be used also for promoting HRQoL.

### Strengths and Limitations of the Study

There are some limitations of the study that should be taken into consideration when interpreting the findings. First, HRQoL was measured with a validated and widely recognized instrument, but retrospective self-reports about sports participation in the childhood were measured with unvalidated questions without information about the frequency of participation. Furthermore, recall bias may have occurred due to the retrospective nature of the questions. However, competitive sport, particularly, represents the most intensive and regular type of sports participation, and it can be assumed that study participants have reported it accurately. Second, although the present self-reported LTPA in adulthood has been validated against fitness (Fogelholm et al., 2006), it has not been validated against, e.g., device-based PA. The main limitation, however, regarding this question is that the variation in the amount of light and moderate PA cannot be assessed. Third, in terms of HRQoL, the differences in scores across the different types and levels of sports performance were quite small and not all necessarily clinically important or meaningful differences. Fourth, in a cross-sectional study with even a retrospective perspective, a causal link between participation in organized sports in childhood and HRQoL in young adulthood cannot be established. There are many other factors, such as different lifestyle or childhood living condition, that may contribute to the HRQoL in adulthood and affect the observed association. For instance, data on family background (e.g., socioeconomic background) in the childhood, which may have a major impact on the possibilities to participate in organized sports at the age of 12 years, were not available. No explanation can be provided if children who participate in organized sports have stronger basis for creating better HRQoL in later life, per se. Fifth, as the sample used in this study—reservists participating in the refresher course—represents a group of healthy young men being physically and mental capable of participating in the course, young men with various health concerns are underrepresented. Future studies with longitudinal study designs are needed to enhance the understanding about the role of sports participation in the childhood for the HRQoL in the life span.

## Conclusion

The present study showed that higher frequency of participation in organized sports, engagement in district-level competitive sports, and performing team, endurance, or extreme sports or sports in informal groups in childhood were all independently associated with better HRQoL in young adulthood. The association was, particularly, found in the mental component of HRQoL and to a lesser extent in the physical component of HRQoL.

In terms of sport types, team and extreme sports seemed to be associated with the mental component of HRQoL and playing yard games with the physical component of HRQoL. Thus, engagement in organized sports—both team-based and individual sports—but also PA in informal groups, such as playing yard games, in childhood may contribute to HRQoL in later life. However, as suggested by Moeijes et al. (2019a), the frequency of sports participation and learning an active lifestyle is more relevant than the form of sports participation. As particular individuals with active childhood sports participation are known to continue their PA also in later life (Telama et al., 1996), children and adolescents should be encouraged to perform any kind of sports activity on a regular basis and support to maintain their active lifestyle as long as possible. Strategies and activities that enhance organized sport participation for children and young people should therefore be promoted. Furthermore, specific strategies should be developed to encourage children and young people with different levels of ability and commitment to participate in organized sport.

## Data Availability Statement

The raw data supporting the conclusions of this article will be made available by the authors, without undue reservation.

## Ethics Statement

The studies involving human participants were reviewed and approved by Ethical committees of the Central Finland Health Care District and the Headquarters of the Finnish Defence Forces (AM5527). The patients/participants provided their written informed consent to participate in this study.

## Author Contributions

The study is part of the Finnish Reservist 2015 Study led by HK. KA-S, HK, AH, TV, MM, and JV contributed to the study design and methodology. JV led the data collection with the assistance of research assistants. KA-S conducted the statistical analysis and led the writing process. All authors provided critical revisions to the manuscript, and accept responsibility for the contents of the article. KA-S had final responsibility for the decision to submit for publication. All authors read and approved the final version submitted.

## Conflict of Interest

The authors declare that the research was conducted in the absence of any commercial or financial relationships that could be construed as a potential conflict of interest.

## References

[B1] Aalto A. M. Aro S. Aro A. Mähönen M. (1995). Rand 36-Item Health Survey 1.0. Suomenkielinen versio terveyteen liittyvästä elämänlaadun kyselystä. Kyselylomake ja käyttöohjeet. Helsinki: Stakes.

[B2] Appelqvist-Schmidlechner K. Vaara J. Häkkinen A. Vasankari T. Mäkinen J. Mäntysaari M.. (2018). Relationships between youth sports participation and mental health in young adulthood among Finnish males. Am. J. Health Prom. 32, 1502–1509. 10.1177/0890117117746336 29268622

[B3] Ashdown-Franks G. Sabiston C. Solomon-Krakus S. O'Loughlin J. (2017). Sport participation in high school and anxiety symptoms in young adulthood. Ment Health Phys. Act. 12, 19–24. 10.1016/j.mhpa.2016.12.001

[B4] Backmand M. H. Kaprio J. Kujala U. M. Sarna S. (2009). Physical activity, mood and the functioning of daily living a longitudinal study among former elite athletes and referents in middle and old age. Arch. Geront. Geriatr. 48, 1–9. 10.1016/j.archger.2007.09.002 17942171

[B5] Biddle S. (2016). Physical activity and mental health: evidence is growing. World Psychiatr. 15, 176–177. 10.1002/wps.20331 27265709 PMC4911759

[B6] Bize R. Johnson J. A. Plotnikof R. C. (2018). Physical activity level and health-related quality of life in the general adult population: a systematic review. Ment. Health Phys. Act. 14, 121–130. 10.1016/j.ypmed.2007.07.017 17707498

[B7] DeSalvo K. Bloser N. Reynolds K. He J. Muntner P. (2006). Mortality prediction with a single general self-rated health question. A meta-analysis. Gen Intern. Med. 21, 267–275. 10.1111/j.1525-1497.2005.00291.x 16336622 PMC1828094

[B8] Dominick K. Ahern F. Gold C. Heller D. (2002). Relationship of health-related quality of life to health care utilization and mortality among older adults. Aging Clin. Exp. Res.. 14, 499–508. 10.1007/BF03327351 12674491

[B9] Dore I. O'Loughlin J. Schnitzer M. Datta G. Fournier L. (2018b). The longitudinal association between the context of physical activity and mental health in early adulthood. Ment Health Phys. Act. 14, 121–130. 10.1016/j.mhpa.2018.04.001

[B10] Dore I. Sabiston C. Sylveste M.-P. Brunet J. (2018a). Years participating in sports during childhood predicts mental health in adolescence: a 5-year longitudinal study. J. Adolesc. Health. 64, 790–796. 10.1016/j.jadohealth.2018.11.024 31122508

[B11] Downward P. Rasciute S. (2011). Does sport make you happy? An analysis of the well-being derived from sports participation. Int. Rev. Appl. Economics. 25, 331–348. 10.1080/02692171.2010.511168

[B12] Eime R. M. Harvey J. T. Brown W. J. Payne W. R. (2010). Does sport club participation contribute to health-related quality of life. Med. Sci. Sport Exer. 42, 1022–1028. 10.1249/MSS.0b013e3181c3adaa 19996991

[B13] Eime R. M. Young J. A. Harvey J. T. Charity M. J. Payne W. R. (2013a). A systematic review of the psychological and social benefits of participation in sport for children and adolescents: informing development of a conceptual model of health through sport. Int. J. Behav. Nutr. Phys. Act. 10:98. 10.1186/1479-5868-10-98 23945179 PMC3751802

[B14] Eime R. M. Young J. A. Harvey J. T. Chariy M. J. Payne W. R. (2013b). A systematic review of the psychological and social benefits of participation in sport for adults: informing development of a conceptual model of health through sport. Int. J. Beh. Nutr. Phys. Act. 10:135. 10.1186/1479-5868-10-135 24313992 PMC4028858

[B15] Ellina P. Middleton N. Lambrinou E. Kouta C. (2019). Investigation of socioeconomic inequalities in health-related quality of life across Europe: a systematic review. Divers Equal Health Care 16, 80–90. 10.36648/2049-5471.16.3.197

[B16] Ferrans C. (2005). Definitions and conceptual models of quality of life, in Outcomes Assessment in Cancer. eds J. Lipscomb, C. Gotay, C. Snyder (Cambridge: Cambridge university), 14–30. 10.1017/CBO9780511545856.002

[B17] Findlay L. C. Coplan R. J. (2008). Come out and play: shyness in childhood and the benefits of organized sports participation. Can J. Behav. Sci. 40, 153–161. 10.1037/0008-400X.40.3.153

[B18] Fogelholm M. Malmberg J. Suni J. Santtila M. Kyröläinen H. Mäntysaari M.. (2006). International physical activity questionnaire: validity against fitness. Med. Sci. Sports Exerc. 38, 753–760 10.1249/01.mss.0000194075.16960.20 16679993

[B19] Houston M. N. Hoch M. C. Hoch J. M. (2016). Health-related quality of life in athletes: a systematic review with meta-analysis. J. Athl. Train. 51, 442–453. 10.4085/1062-6050-51.7.03 27258942 PMC5076283

[B20] Immonen T. Brymer E. Orth D. Davids K. Feletti F. Liukkonen J.. (2017). Understanding action and adventure sports participation – An ecological dynamics perspective. Sports Med. 3:18. 10.1186/s40798-017-0084-1 28447331 PMC5406377

[B21] Jewett R. Sabiston C. Brunet J. O'Loughlin E. Scarapicchia T. O'Loughlin J. (2014). School sport participation during adolescence and mental health in early adulthood. J. Adolesc Health. 55, 640–644. 10.1016/j.jadohealth.2014.04.018 24928804

[B22] Lubans D. Richards J. Hillman C. Faulkner G. Beauchamp M. Nilsson M.. (2016). Physical activity for cognitive and mental health in youth: a systematic review of mechanisms. Pediatrics 138:e20161642. 10.1542/peds.2016-1642 27542849

[B23] Marker A. M. Steele R. G. Noser A. E. (2018). Physical activity and health-related quality of life in children and adolescents: a systematic review and meta-analysis. Health Psych. 37, 893–903. 10.1037/hea0000653 30234348

[B24] Merkel D. (2013). Youth sport: positive and negative impact on young athletes. J. Sports Med. 4, 151–160. 10.2147/OAJSM.S33556 24379720 PMC3871410

[B25] Moeijes J. van Busschbach J. T. Bosscher R. J. Twisk J. W. R. (2019a). Sports participation and health-related quality of life: a longitudinal observational study in children. Qual. Life Res. 28, 2453–2469. 10.1007/s11136-019-02219-4 31161332 PMC6698265

[B26] Moeijes J. van Busschback J. T. Wieringa T. H. Kone J. Bosscher R. J. Twisk J. W. R. (2019b). Sports participation and health-related quality of life in children: results of a cross-sectional study. Health Qual. Life Outcomes. 27. 10.1186/s12955-019-1124-y 30987637 PMC6466683

[B27] Roberts L. Jones G. Brooks R. (2018). Why do you ride? A characterization of mountain bikers, their engagement methods, and perceived links to mental health and well-being. Front Psychol. 19:1642. 10.3389/fpsyg.2018.01642 30283372 PMC6156442

[B28] Rutten A. Abu-Omar K. Morgan A. Groce N. Stuart J. (2008). Research note: social catalysts in health promotion implementation. J. Epidemiol. Community Health. 62, 560–565. 10.1136/jech.2007.062190 18477757

[B29] Sabiston C. M. Jewett R. Ashdown-Franks G. Belanger M. Brunet J. O'Loughlin E.. (2015). Number of years of team and individual sport participation during adolescence and depressive symptoms in early adulthood. J. Sport Exerc. Psychol. 38, 105–110. 10.1123/jsep.2015-0175 27018562

[B30] Shaikh S. I. Ansari M. A. Sheeraz B. Kalhoro Z. A. (2016). Quality of life and mental health among university students: a comparison of sports participants and non-participants. Shields 11, 1–11. Available online at: https://sujo-old.usindh.edu.pk/index.php/THE-SHIELD/article/view/3282/2380

[B31] Sneddden T. R. Scerpella J. Klethermes S. A. Norman R. Blyholder L. Sanfilippo J.. (2018). Sport and physical activity level impacts health-related quality of life among collegiate students. Am. J. Health Prom. 33, 675–682. 10.1177/0890117118817715 30586999 PMC7213817

[B32] Snyder A. R. Martinez J. C. Bay R. C. Parsons J. T. Sauers E. L. Valovich McLeod T. C. (2010). Health-related quality of life differs between adolescent athletes and adolescent nonathletes. J. Sport Rehabil. 19, 237–248. 10.1123/jsr.19.3.237 20811075

[B33] Sorensen S. C. Romano R. Scholefield R. M. Martin B. E. Gordon J. E. Azen S. P.. (2014). Holistic life-span health outcomes among elite intercollegiate student-athletes. J. AthlTrain. 49, 684–695. 10.4085/1062-6050-49.3.18 25117874 PMC4208874

[B34] Stracciolini A. Amar-Dolan L. Howell D. Alex T. Berkner P. Sandstrom N.. (2018). Female sport participation effect on long-term health-related quality of life. Clin. J. Sport Med. 30:526–32. 10.1097/JSM.0000000000000645 30095508

[B35] Telama R. Leskinen E. Yang X. (1996). Stability of habitual physical activity and sports participation: a longitudinal tracking study. Scand. J. Med. Sci. Sports. 6, 371–378. 10.1111/j.1600-0838.1996.tb00109.x 9046549

[B36] Vella S. A. Cliff D. P. Magee C. A. Okely A. D. (2014). Sports participation and parent-reported health-related quality of life in children: longitudinal associations. J. Pediatr. 164, 1460–1474. 10.1016/j.jpeds.2014.01.071 24657117

[B37] Wagnsson S. Lindwall M. Gustafsson H. (2014). Participation in organized sport and self-esteem across adolescence: the mediating role of perceived sport competence. J. Sport Exerc. Psychol. 36, 584–594. 10.1123/jsep.2013-0137 25602141

[B38] Warburton D. Bredin S. (2017). Health benefits of physical activity and systematic review of current systematic reviews. Curr. Opin. Cardiol. 32, 541–556. 10.1097/HCO.0000000000000437 28708630

[B39] Wu X. Y. Han L. H. Zhang J. H. Luo S. Hu J. W. Sun K. (2017). The influence of physical activity, sedentary behavior on health-related quality of life among the general population of children and adolescents: a systematic review. PLoS ONE. 12:187668. 10.1371/journal.pone.0187668 29121640 PMC5679623

